# Purine Metabolites and Carnitine Biosynthesis Intermediates Are Biomarkers for Incident Type 2 Diabetes

**DOI:** 10.1210/jc.2019-00822

**Published:** 2019-07-24

**Authors:** Filip Ottosson, Einar Smith, Widet Gallo, Céline Fernandez, Olle Melander

**Affiliations:** Department of Clinical Sciences, Lund University, Malmö, Sweden

## Abstract

**Context:**

Metabolomics has the potential to generate biomarkers that can facilitate understanding relevant pathways in the pathophysiology of type 2 diabetes (T2DM).

**Methods:**

Nontargeted metabolomics was performed, via liquid chromatography–mass spectrometry, in a discovery case-cohort study from the Malmö Preventive Project (MPP), which consisted of 698 metabolically healthy participants, of whom 202 developed T2DM within a follow-up time of 6.3 years. Metabolites that were significantly associated with T2DM were replicated in the population-based Malmö Diet and Cancer–Cardiovascular Cohort (MDC-CC) (N = 3423), of whom 402 participants developed T2DM within a follow-up time of 18.2 years.

**Results:**

Using nontargeted metabolomics, we observed alterations in nine metabolite classes to be related to incident T2DM, including 11 identified metabolites. N2,N2-dimethylguanosine (DMGU) (OR = 1.94; *P* = 4.9e-10; 95% CI, 1.57 to 2.39) was the metabolite most strongly associated with an increased risk, and beta-carotene (OR = 0.60; *P* = 1.8e-4; 95% CI, 0.45 to 0.78) was the metabolite most strongly associated with a decreased risk. Identified T2DM-associated metabolites were replicated in MDC-CC. Four metabolites were significantly associated with incident T2DM in both the MPP and the replication cohort MDC-CC, after adjustments for traditional diabetes risk factors. These included associations between three metabolites, DMGU, 7-methylguanine (7MG), and 3-hydroxytrimethyllysine (HTML), and incident T2DM.

**Conclusions:**

We used nontargeted metabolomics in two Swedish prospective cohorts comprising >4000 study participants and identified independent, replicable associations between three metabolites, DMGU, 7MG, and HTML, and future risk of T2DM. These findings warrant additional studies to investigate a potential functional connection between these metabolites and the onset of T2DM.

## Teaser

Plasma levels of metabolites DMGU, 7MG, and HTML are associated with incident T2DM in two prospective cohorts comprising >4000 patients.

Type 2 diabetes (T2DM) is a complex disease characterized by hyperglycemia as a result of insulin resistance and insulin insufficiency. Describing the early metabolic changes in T2DM, which often occur years before clinical manifestation, could be key to understanding the pathophysiology of T2DM. Metabolomics has emerged as a powerful tool to describe metabolic changes in T2DM by searching for candidate metabolite biomarkers in blood samples from large prospective cohorts (1, 2). By using this approach, metabolomic studies have revealed that alterations of several metabolites and metabolic pathways are linked to risk of T2DM, including branched-chain amino acids (3–8), aromatic amino acids (3, 9), acylcarnitines (4), glutamate (4, 10), dimethylguanidine valerate (11), and phospholipids (9).

Although the discovered candidate metabolite biomarkers have shown great potential, the bulk of the studies performed in large prospective cohorts have used a targeted metabolomics approach, measuring only a limited portion of the metabolome in a hypothesis-driven approach. Only in a small number of studies has a broader nontargeted metabolomics approach been applied to identify promising candidate biomarkers for T2DM (6, 7, 12). However, there are differences between studies in terms of pinpointing key metabolites predicting future T2DM, which could be driven by differences in study population, analytical methods, and statistical analysis. Given these inconsistencies, further studies are needed to provide the medical community with a more comprehensive description of the metabolic alterations that precede T2DM.

In our previous studies using targeted metabolomics, we have identified branched-chain and aromatic amino acids and glutamate to be strongly related to body mass index (BMI) (13) and incidence of T2DM (4). In this study our aim was to use a nontargeted metabolomics approach to identify plasma metabolite biomarkers for the incidence of T2DM in two Swedish prospective cohorts. Candidate metabolite biomarkers were identified in the discovery cohort Malmö Preventive Project (MPP) (N = 698) and replicated in the independent population-based cohort Malmö Diet and Cancer–Cardiovascular Cohort (MDC-CC) (N = 3423).

## Methods

### Study populations

MPP is a population-based prospective cohort of 33,346 participants, enrolled between 1974 and 1992. Between 2002 and 2006, 18,240 participants (65 to 80 years old) were reexamined for cardiometabolic risk factors, and overnight-fasting EDTA plasma was collected and stored at −80°C for later analyses. This reexamination forms the baseline of the current prospective analysis. A case-cohort subset from the reexamination of MPP was used as a discovery cohort and has been described in more detail previously (4). Briefly, the subcohort consisted of 698 participants who were free of cardiometabolic disease at baseline, but it included 202 participants who developed incident T2DM over an average follow-up time of 6.3 years.

Malmö Diet and Cancer (MDC) is a population-based cohort designed to study the epidemiology of carotid artery disease, with participants being enrolled between 1991 and 1996. Among the 5405 participants who came fasted to the random subsample called the MDC-CC, citrate plasma was obtained from 3799 participants for analysis. The 3799 subjects included are compared with participants with missing plasma samples in an online repository (14). After exclusion of participants with reported diabetes at baseline or with fasting blood glucose >6.0 mmol/L (corresponding to plasma glucose of 7.0 mmol/L), 3423 participants remained. During an average follow-up time of 18.2 years, 402 of these 3423 nondiabetic participants developed incident T2DM.

### Endpoint definition and biochemical measurements

T2DM was defined as a fasting plasma glucose >7.0 mmol/L, a history of physician diagnosis of T2DM, being on antidiabetic medication, or having been registered in local or national Swedish diabetes registries (15). Measurements of fasting total cholesterol, high-density lipoprotein (HDL) cholesterol, triglycerides, and glucose were made according to standard procedures at the Department of Clinical Chemistry at Malmö University Hospital. Systolic blood pressure was measured with a mercury-column sphygmomanometer after 10 minutes of rest in the supine position.

### Analytical procedure

Profiling of plasma metabolites was performed with a UPLC-QTOF-MS System (1290 LC, 6550 MS; Agilent Technologies, Santa Clara, CA) and has previously been described in detail (16). Briefly, plasma samples stored at −80°C were thawed and extracted by addition of six volumes of extraction solution. The extraction solution consisted of 80:20 methanol/water. Extracted samples were separated on an Acquity UPLC BEH Amide column (1.7 μm, 2.1 * 100 mm; Waters Corporation, Milford, MA). Quality control samples, consisting of pooled plasma, were injected into every eight analytical sample to ensure high repeatability and to enable normalization of metabolite measurements.

### Data processing and metabolite selection

Metabolite feature extraction was performed with an Agilent Mass Hunter Profinder B.06.00 in pooled plasma samples from the MPP. Metabolite features that were present in ≥80% of the pooled samples (N = 1908) were extracted from analytical samples and quality control samples. Normalization was performed via metabolite measurements in the quality control samples. A low-order nonlinear locally estimated smoothing function was fitted to the measurements of each metabolite feature in the quality control samples. These functions were used to create correction curves, to which each metabolite feature measurement in the analytical samples were normalized (17).

Only metabolite features that had a coefficient of variation <20% in the repeated injections of quality control samples and were measured in >90% of the participants in MPP were selected for statistical analysis (N = 1025) to ensure high repeatability of studied metabolite features. Metabolites that were previously investigated in the same study material, via a targeted metabolomics method, were excluded from the analysis (4). The study workflow is presented in an online repository (14).

### Metabolite annotation and identification

We performed metabolite annotation for metabolite features associated with T2DM by matching spectra and retention times against an in-house metabolite library and publicly available databases. Annotations followed the Metabolomics Standard Initiative guidelines (18) by defining annotation confidence levels (level 1 to 4) to each metabolite feature. Level 1 confidence (identified metabolite) required accurate mass-over-charge ratio (*m*/*z*), retention time, and fragmentation spectra as synthetic standards in the in-house metabolite library. Level 2 confidence (annotated metabolite) required accurate *m*/*z* and fragmentation in a public database. Level 3 confidence (annotated metabolite class) required *m*/*z*, fragmentation spectra, or retention time to be consistent with previous data on metabolites from a certain class. Level 4 confidence consists of unknown metabolite features. Metabolite identifications are presented in an online repository (14). Metabolite standards were either purchased or derived from synthetic standards purchased from Toronto Research Chemicals (New York, ON, Canada).

### Statistical analysis

The associations between plasma levels of metabolite features and incident T2DM in MPP were investigated via logistic regression models, adjusted for age and sex. In secondary analyses in MPP, logistic regression models were adjusted for BMI and fasting glucose levels. Identified T2DM-associated metabolites were replicated in the population-based MDC-CC, via Cox proportional hazards models. Primary Cox proportional hazards models were adjusted for age and sex, and the secondary models were additionally adjusted for BMI and fasting glucose. In MPP, intermetabolite correlations were investigated via Spearman correlation tests. Correlations between annotated metabolites and T2DM risk factors were analyzed via partial Spearman correlations, adjusted for age and sex. All statistical analyses were performed in R version 3.4.3.

## Results

The baseline characteristics of the two prospective cohorts, MPP (case-cohort) and MDC-CC (population-based), can be found in Table 1. Several cardiometabolic risk factors were different between incident T2DM and participants who remained free from T2DM during follow-up.

**Table 1.** Characteristics of Participants in the MDC and MPP

	MPP			MDC		
Trait	Controls (N = 496)	Incident T2DM (N = 202)	*P*	Cohort Without T2DM (N = 3021)	Incident T2DM (N = 402)	*P*
Age, y	68.7 (±5.9)	69.3 (±5.7)	0.31	57.5 (±6.0)	57.8 (±5.8)	0.25
Sex, % female	37.2	31.4	0.16	61.0	55.0	0.023
BMI, kg/m^2^	26.5 (±4.2)	29.2 (±4.8)	<0.001	25.1 (±3.5)	27.4 (±4.6)	<0.001
Fasting glucose, mmol/L	5.4 (±0.5)	6.0 (±0.6)	<0.001	4.9 (±0.4)	5.2 (±0.4)	<0.001
Systolic blood pressure, mm HG	143 (±21)	149 (±20)	0.0030	140 (±19)	146 (±19)	<0.001
HDL cholesterol, mmol/L	1.42 (±0.43)	1.27 (±0.38)	<0.001	1.43 (±0.31)	1.28 (±0.37)	<0.001
LDL cholesterol, mmol/L	3.7 (±0.9)	3.7 (±1.0)	0.79	4.13 (±1.0)	4.33 (±1.0)	<0.001
Triglycerides, mmol/L	1.2 (±0.6)	1.4 (±0.7)	<0.001	1.22 (±1.2)	1.53 (±1.5)	<0.001

Differences in the baseline characteristics between participants with incident T2DM and participants without incident T2DM were investigated via two-sample *t* test (continuous variables) and Pearson *χ*^2^ test (sex).

### Nontargeted metabolomics in MPP reveals several metabolite classes connected to future risk of T2DM

**Figure 1.**
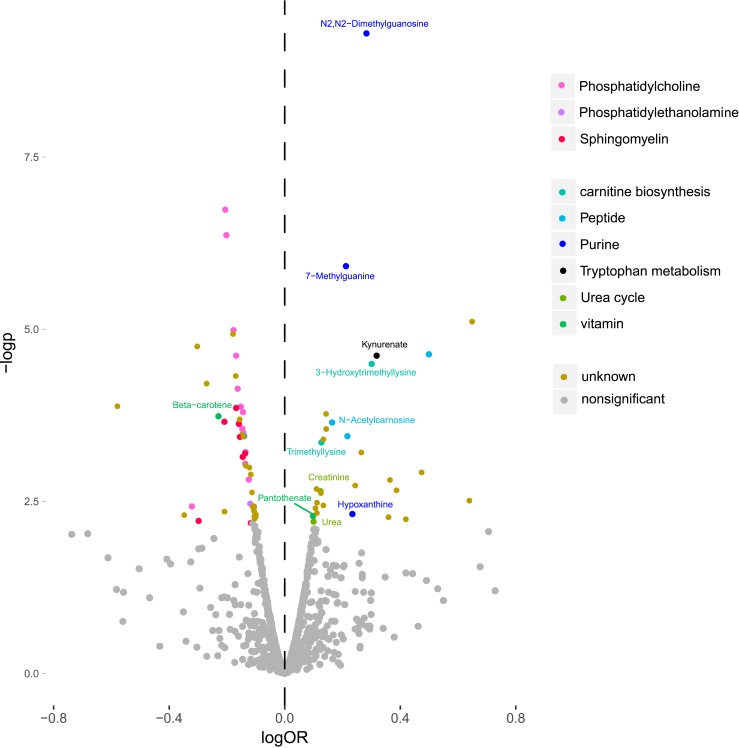
Metabolite features from different metabolite classes associated with incident T2DM in the MPP (N = 698). logOR is the 10 log of the OR calculated from logistic regression models, and –logp is the negative 10 log of the *P* value calculated from the logistic regression models. Metabolite features with an annotation confidence at level 4 are marked as unknown. Metabolite features with annotation confidence of 2 or 3 are colored according to their metabolite class, and metabolites with annotation confidence 1 are additionally named in the figure.

**Figure 2.**
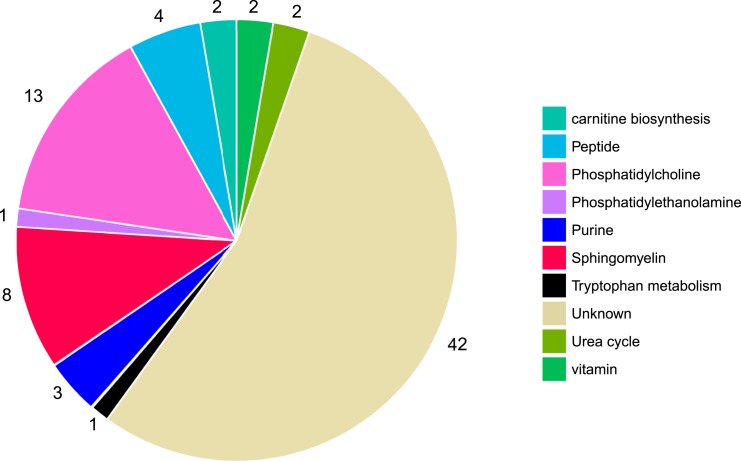
T2DM-associated metabolites belong to several metabolite classes. Metabolites that are defined as being at a level 4 metabolite annotation confidence are characterized as unknown (N = 42). Metabolites with an annotation confidence of ≥3 are characterized according to their metabolite class (N = 36).

Among the 1025 metabolite features that were measured in MPP, 78 were significantly associated with incident T2DM (false discovery rate <0.05), via age- and sex-adjusted logistic regression models (Fig. 1). Metabolite features were annotated if they reached or exceeded level 3 of annotation confidence (36 T2DM-associated metabolite features). In total, 22 of the T2DM-associated metabolite features could be annotated as lipids, belonging to any of the three phospholipid classes: sphingomyelin, phosphatidylethanolamine, or phosphatidylcholine. All phospholipids were associated with a lower risk of future T2DM, and most nonlipid features were associated with an increased risk. The nonlipid metabolites belonged to several metabolite classes, including peptides, purine metabolism, carnitine biosynthesis, urea cycle, and tryptophan metabolism (Fig. 2).

**Figure 3.**
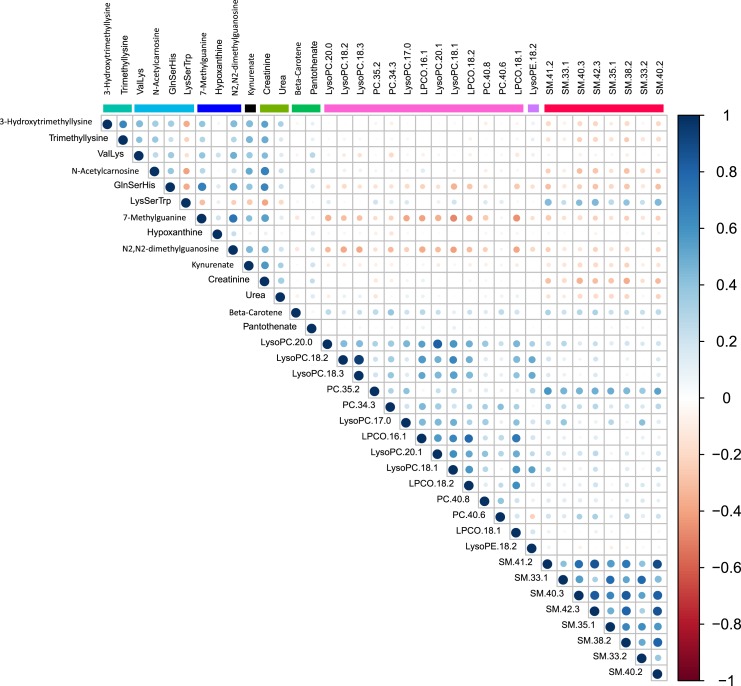
Correlation matrix of intermetabolite correlations in the MPP (N = 698). Correlations are Spearman *ρ* coefficients.

There were strong correlations between metabolites within several metabolite classes (Fig. 3). Particularly strong intraclass correlations were observed in sphingomyelins, phosphatidylcholines, purine metabolites, and carnitine biosynthesis.

**Figure 4.**
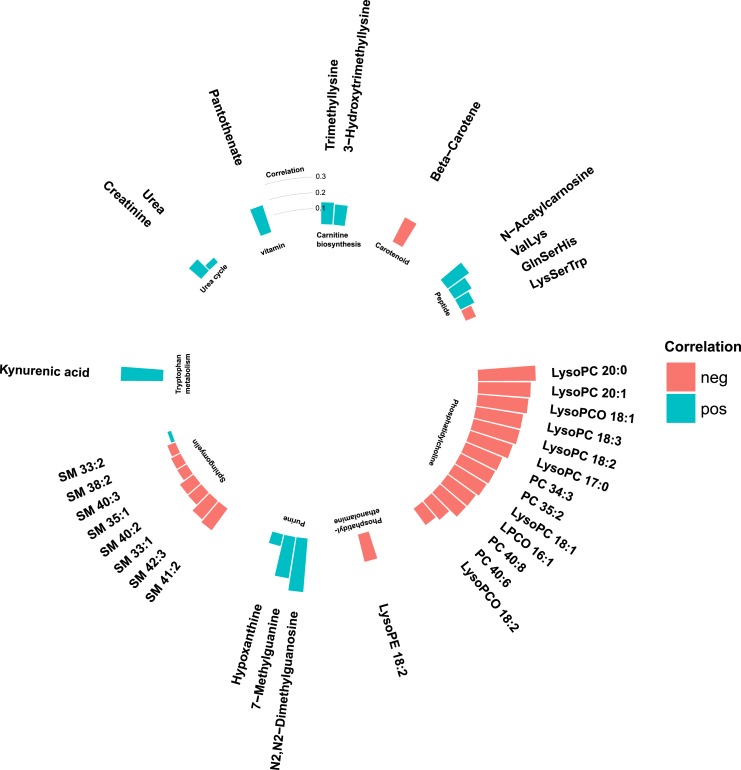
Partial Spearman correlations between annotated T2DM-associated metabolites and BMI in the MPP (N = 698). Positive correlations are marked in blue and negative correlations in red.

**Figure 5.**
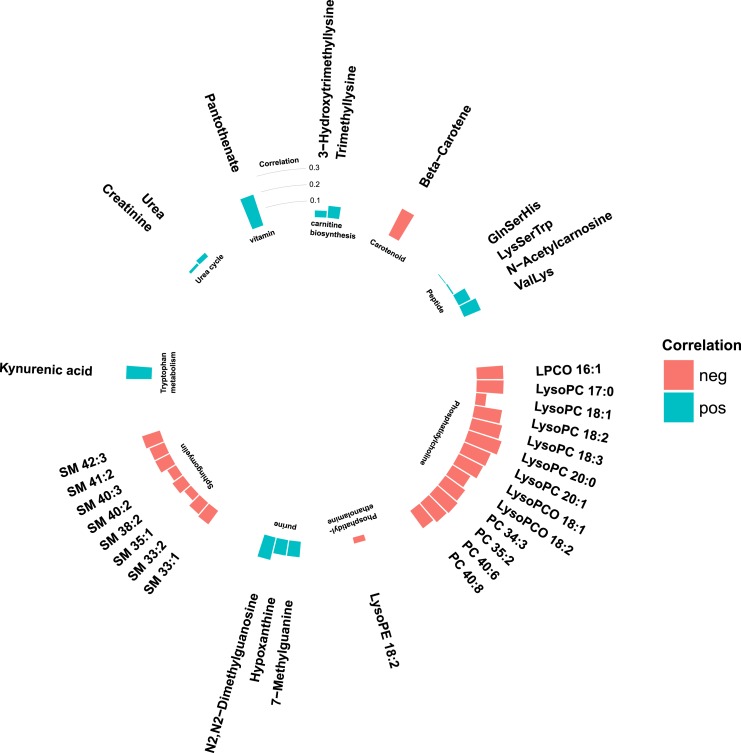
Partial Spearman correlations between annotated T2DM-associated metabolites and fasting glucose levels in the MPP (N = 698). Positive correlations are marked in blue and negative correlations in red.

Phospholipids were in general negatively correlated with fasting glucose levels and BMI (Figs. 4 and 5), where particularly strong correlations were found between BMI and phosphatidylcholines and phosphatidylethanolamines. Most nonlipid metabolites showed significant but weak or moderate correlations with BMI (Fig. 4) and fasting glucose (Fig. 5). Two of the purine metabolites [N2,N2-dimethylguanosine (DMGU) and 7-methylguanine (7MG)] and the tryptophan metabolite (kynurenate) were strongly correlated with BMI. One vitamin (pantothenate) was strongly correlated with elevated fasting glucose. There were also strong correlations between several diabetes-associated metabolites and fasting levels of HDL cholesterol and triglycerides. In general, the phospholipids, purine metabolites, and carnitine biosynthesis intermediates were positively correlated with HDL cholesterol and negatively correlated with triglycerides. Most diabetes-associated metabolites were less correlated with systolic blood pressure and fasting levels of low-density lipoprotein (LDL) cholesterol (14). All correlations between metabolites and T2DM risk factors are found in an online repository (14).

### Eleven identified metabolites associated with incident T2DM in MPP

Among 11 metabolite features reaching a level 1 annotation confidence (Fig. 1), three metabolites were related to purine metabolism and included the two metabolites most strongly associated with incident T2DM, DMGU (OR = 1.94; 95% CI, 1.57 to 2.39; *P* = 4.9e-10) and 7MG (OR = 1.65; 95% CI, 1.35 to 2.01; *P* = 1.2e-6), and hypoxanthine (OR = 1.73, 95% CI, 1.18 to 2.54, *P* = 4.7e-3). The other metabolite classes with T2DM-associated metabolites were carnitine biosynthesis [trimethyllysine and 3-hydroxytrimethyllysine (HTML)], urea cycle (creatinine and urea), tryptophan metabolism (kynurenate), peptides (*N*-acetylcarnosine), and vitamins (pantothenate and beta-carotene) (Table 2). All 11 identified T2DM-associated metabolites were higher in incident T2DM cases except beta-carotene, which was lower.

**Table 2.** Associations Between Plasma Metabolite Levels and Incidence of T2DM in the MPP (N = 698) and the MDC-CC (N = 3423)

	MPP (N = 698)		MDC-CC (N = 3423)	
Metabolite	OR	*P*	HR	*P*
DMGU	1.94 (1.57–2.39)	4.9e-10	1.63 (1.35–1.98)	3.2e-7
7MG	1.65 (1.35–2.01)	1.2e-6	1.49 (1.22–1.83)	1.1e-4
Kynurenate	2.10 (1.49–2.97)	2.3e-5	1.31 (1.16–1.48)	1.1e-5
HTML	2.02 (1.45–2.82)	3.1e-5	1.50 (1.19–1.87)	5.1e-4
Beta-carotene	0.60 (0.45–0.78)	1.8e-4	0.68 (0.60–0.78)	2.3e-8
*N*-Acetylcarnosine	1.48 (1.20–1.82)	2.2e-4	1.27 (1.11–1.45)	4.7e-4
TML	1.35 (1.14–1.60)	4.2e-4	1.20 (1.07–1.34)	2.0e-3
Creatinine	1.35 (1.11–1.63)	2.2e-3	1.07 (0.94–1.21)	0.28
Hypoxanthine	1.73 (1.18–2.54)	4.7e-3	1.24 (1.11–1.38)	1.5e-4
Pantothenate	1.27 (1.07–1.49)	5.0e-3	1.13 (1.01–1.27)	0.027
Urea	1.27 (1.07–1.51)	6.1e-3	1.18 (1.06–1.31)	2.7e-3

ORs and HRs are expressed per SD increment of plasma metabolite and calculated from logistic regression models. Regression models are adjusted for age and sex.

### T2DM associated metabolites are replicable in MDC

The associations with incident T2DM for the 11 identified metabolites were replicated in the independent population-based cohort MDC-CC. All metabolites except creatinine were associated with incident T2DM in the replication cohort. Beta-carotene was the metabolite most strongly associated with incident T2DM in MDC-CC [hazard ratio (HR) = 0.68; 95% CI, 0.60 to 0.78; *P* = 2.3e-8], followed by the two purine metabolites DMGU (HR = 1.63; 95% CI, 1.35 to 1.98; *P* = 3.2e-7) and 7MG (HR = 1.49; 95% CI, 1.22 to 1.83; *P* = 1.1e-4) (Table 2).

### Four metabolites are independent and replicable candidate biomarkers for incident T2DM

Because overweight and hyperglycemia are key features of prediabetes and were correlated with many of the T2DM-associated metabolites, all regression models were additionally adjusted for BMI and fasting glucose. After the adjustments, the associations between metabolite levels and incident T2DM were generally attenuated in both cohorts, but seven associations remained significant in MPP and six in MDC-CC (14). Four metabolites, DMGU, 7MG, HTML, and urea, remained significantly associated with incident T2DM after multivariable adjustments in both cohorts, indicating that the metabolites are associated with incident T2DM independently of traditional diabetes risk factors (Table 3). The T2DM associations for the metabolites beta-carotene and hypoxanthine were attenuated after risk factor adjustments in both cohorts, but they remained significant only in MDC-CC. Trimethyllysine and *N*-acetylcarnosine remained significantly associated with incident T2DM after risk factor adjustment only in the MPP cohort. Kynurenate and pantothenate were not independently associated with incident T2DM in any of the cohorts. When additional adjustments for systolic blood pressure and fasting levels of triglycerides were performed, HDL and LDL cholesterol, DMGU, 7MG, and HTML remained significantly associated in both cohorts (Table 3).

**Table 3.** Associations Between Plasma Metabolite Levels and Incidence of T2DM in the MPP (N = 698) and the MDC-CC (N = 3423)

	MPP (N = 698)				MDC-CC (N = 3423)			
	Model 1		Model 2		Model 1		Model 2	
Metabolite	OR	*P*	OR	*P*	HR	*P*	HR	*P*
DMGU	1.67 (1.31–2.12)	3.6e-5	1.54 (1.20–1.99)	8.0e-4	1.28 (1.05–1.55)	0.012	1.28 (1.05–1.55)	0.014
7MG	1.49 (1.19–1.87)	5.9e-4	1.41 (1.12–1.79)	4.1e-3	1.25 (1.02–1.53)	0.035	1.24 (1.01–1.52)	0.043
HTML	1.50 (1.19–1.87)	5.1e-4	1.82 (1.25–2.65)	1.7e-3	1.41 (1.12–1.77)	3.0e-3	1.42 (1.13–1.79)	2.8e-3
Urea	1.23 (1.01–1.49)	0.036	1.19 (0.98–1.45)	0.072	1.24 (1.02–1.26)	0.024	1.14 (1.02–1.27)	0.020

ORs and HRs are expressed per SD increment of plasma metabolite and calculated from logistic regression models. Regression model 1 is adjusted for age, sex, fasting glucose, and BMI. Model 2 is additionally adjusted for systolic blood pressure and fasting levels of triglycerides, HDL, and LDL cholesterol.

## Discussion

In this study we present three diabetes-associated metabolites, DMGU, 7MG, and HTML, all of which were associated with future risk of T2DM, independent of traditional diabetes risk factors, in two Swedish prospective cohorts comprising >4000 study participants.

### Alterations in the purine metabolism is connected to diabetes incidence

We identified three associations between purine metabolites and incident T2DM (DMGU, 7MG, and hypoxanthine). DMGU and 7MG are both important constituents of the tRNA molecule, stabilizing its cloverleaf structure (19), and circulating tRNA fragments have been associated with oxidative stress (20). Although neither of the metabolites has previously been associated with diabetes risk, DMGU has been shown to be strongly connected to BMI (21) and suggested to be a biomarker for chronic kidney disease (22) and pulmonary arterial hypertension (23). 7MG was recently found to be associated with cardiovascular and all-cause mortality in a population of male Finnish smokers (24). Despite the strong correlation with BMI, which was also confirmed in this study, the association between DMGU and T2DM was independent of traditional diabetes risk factors, indicating that the association with incident diabetes was not driven merely by obesity. Hypoxanthine is a precursor of uric acid, which has been described as highly correlated to BMI (21) and has been suggested to be involved in the pathogenesis of T2DM (25). Hypoxanthine is converted to uric acid in a two-step reaction catalyzed by xanthine oxidase, the enzymatic activity of which has been associated with an increased risk of future diabetes (26). Given the metabolic relatedness between hypoxanthine and uric acid, it is possible that the association between hypoxanthine and incident T2DM reflects the same physiological mechanisms that drive the association between uric acid and incident T2DM. Interestingly, plasma levels of hypoxanthine were only weakly correlated to levels of the other two diabetes-related purines 7MG and DMGU, whose levels were strongly correlated. This indicates that although the three metabolites belong to the same overall metabolite class, their relation to incident T2DM might be mediated through two different mechanisms.

### Carnitine biosynthesis, a diabetes-related pathway

In this study, we present associations between incident diabetes and the carnitine biosynthesis intermediates, trimethyllysine (TML) and HTML. TML has previously been shown to predict future risk of T2DM in patients with suspected stable angina pectoris (27) and to be associated with incident cardiovascular disease and cardiovascular mortality (28). This study presents associations between HTML and incident T2DM, further reinforcing the connection between carnitine biosynthesis and T2DM risk. Carnitine is synthesized from protein-derived lysine residues, in a series of reactions in the carnitine biosynthesis pathway involving the hydroxylation of TML to HTML by TML hydroxylase in the mitochondria (29). The positive association of both TML and HTML with incident T2DM indicates that an increased flux through this pathway, leading to increased formation of carnitine, could be connected to an increased risk of T2DM. Because plasma levels of carnitine and acylcarnitines have been associated with incident T2DM (30), the associations between T2DM incidence and TML or HTML levels might be driven by an increased availability of circulating carnitine. Circulating carnitine levels depend on both endogenous synthesis and dietary intake; 75% of the carnitine in humans is derived from the diet (31). Additional studies are needed to evaluate whether the connection between increased levels of TML and HTML and T2DM incidence is driven by increased carnitine production, or whether increased TML or HTML levels, or both, are primary etiological factors in the pathogenesis of T2DM.

### Alterations in a broad range of metabolite classes are related to incident diabetes

In this study, we observed alterations in at least nine different metabolite classes, including both polar and lipid metabolites. Although not all alterations discussed above were independently associated with incident T2DM, they highlight the complex metabolic disturbances that precede T2DM.

We have shown that plasma kynurenate levels are associated with future risk of T2DM. However, kynurenate has recently been connected to several features of the metabolic syndrome, including obesity (21, 32) and insulin resistance (33). Because the observed association with incident T2DM in our study was clearly attenuated after BMI adjustment and kynurenate was strongly correlated with BMI, the association is probably driven by obesity.

Beta-carotene was found to be associated with incident T2DM in both MPP and MDC, and previous work has shown that beta-carotene levels predict future T2DM in a prospective cohort (34). Because beta-carotene is a precursor for vitamin A, abundantly available in vegetables, it was previously proposed to be a link between vegetable intake and decreased diabetes risk. This theory was refuted by results from a randomized controlled trial (35) showing that beta-carotene intake did not influence future risk of T2DM and by a Mendelian randomization study showing that beta-carotene–elevating genetic variants were not associated with incident T2DM (36).

### Limitations and strengths

The current study has several limitations and strengths. Although we reveal three metabolite biomarkers for future T2DM, the observational nature of the study design makes us unable to prove a causal link to diabetes development. The ability to replicate our findings in two independent prospective cohorts, with differences in baseline age, sex distribution, risk factor profiles, and follow-up times, is a major strength that increases the generalizability of the findings. However, the different study designs in MPP and MDC required different regression models to study incidence of T2DM and coronary artery disease. Therefore, effect estimates cannot be compared between the studies. For end-point retrieval, rather than reexamination with glucose measurements, we relied on registry-based follow-up for incident diabetes, which has the advantage of no loss to follow-up and the disadvantage of not capturing people who do not come in contact with the health care system. In this study T2DM was assessed based on fasting plasma glucose and physician reports in local and national registries. Thus, additional data on glycated hemoglobin levels and 120-minute measurements from oral glucose tolerance would have been beneficial.

## Conclusion

The carnitine biosynthesis intermediate HTML and the purine metabolites DMGU and 7MG are markers for increased risk of future T2DM. This finding warrants future studies to investigate a potential functional connection between these metabolites and the onset of T2DM.

## Abbreviations:

7MG: 7-methylguanine
BMI: body mass index
DMGU: N2,N2-dimethylguanosine
HDL: high-density lipoprotein
HR: hazard ratio
HTML: 3-hydroxytrimethyllysine
LDL: low-density lipoprotein
MDC: Malmö Diet and Cancer
MDC-CC: Malmö Diet and Cancer–Cardiovascular Cohort
MPP: Malmö Preventive Project
T2DM: type 2 diabetes
TML: trimethyllysine
